# Patient-specific effects of soluble factors from *Staphylococcus aureus* and *Staphylococcus epidermidis* biofilms on osteogenic differentiation of primary human osteoblasts

**DOI:** 10.1038/s41598-021-96719-4

**Published:** 2021-08-26

**Authors:** Jutta Tübel, Elisabeth Maier, Magdalena Jegen, Carmen Marthen, Andreas Obermeier, Alexander T. Haug, Jochen Schneider, Rainer Burgkart

**Affiliations:** 1 School of Medicine, Klinikum rechts der Isar, Klinik für Orthopädie und Sportorthopädie, Technische Universität München, Munchen, Germany; 2 School of Medicine, Klinikum rechts der Isar, Klinik und Poliklinik für Innere Medizin II, Technische Universität München, Munchen, Germany

**Keywords:** Infectious diseases, Bacterial infection, Gene expression, Microbiology, Medical research

## Abstract

Due to the frequency of biofilm-forming *Staphylococcus aureus* and *Staphylococcus epidermidis* in orthopedics, it is crucial to understand the interaction between the soluble factors produced by prokaryotes and their effects on eukaryotes. Our knowledge concerning the effect of soluble biofilm factors (SBF) and their virulence potential on osteogenic differentiation is limited to few studies, particularly when there is no direct contact between prokaryotic and eukaryotic cells. SBF were produced by incubating biofilm from *S. aureus* and *S.* *epidermidis* in osteogenic media. Osteoblasts of seven donors were included in this study. Our results demonstrate that the detrimental effects of these pathogens do not require direct contact between prokaryotic and eukaryotic cells. SBF produced by *S. aureus* and *S. epidermidis* affect the metabolic activity of osteoblasts. However, the effect of SBF derived from *S. aureus* seems to be more pronounced compared to that of *S. epidermidis*. The influence of SBF of *S. aureus* and *S.* *epidermidis* on gene expression of *COL1A1, ALPL, BGLAP, SPP1, RUNX2* is bacteria-, patient-, concentration-, and incubation time dependent. Mineralization was monitored by staining the calcium and phosphate deposition and revealed that the SBF of *S. epidermidis* markedly inhibits calcium deposition; however, *S.* *aureus* shows a less inhibitory effect. Therefore, these new findings support the hypotheses that soluble biofilm factors affect the osteogenic processes substantially, particularly when there is no direct interaction between bacteria and osteoblast.

## Introduction

65% of patients with bacterial infections are caused by biofilm-forming bacteria^1,2^. In orthopedics, biofilm forming *Staphylococcus aureus* (*S.* *aureus*) and *Staphylococcus epidermidis (S. epidermidis)* are known as the predominant species, responsible for the most common bone infections, e.g. implant-associated infections and osteomyelitis (66%)^3^. Once invaded, these pathogens cause inflammation and thereby, damage bone tissue. This process may lead to loosening of the implant by osteolysis induced at the implant-bone interface. Prosthesis loosening causes pain, hampers function of the limb affected and is one of the main reasons for revision surgery^4–6^.

Bone remodeling is a complex and dynamic cell process regulated by a “basic multicellular unit”^7^. The coordinated activities of osteoclasts and osteoblasts allow for bone’s capacity for repairing itself, e.g. after small fractures or accumulation of microcracks. Osteoclasts, originated from hematopoietic lineage, are bone-resorbing cells responsible for the breakdown of bone material^8^. Osteoblasts, deriving from mesenchymal stem cells, are bone-forming cells that secrete extracellular matrix proteins and regulate subsequent matrix mineralization^9–11^. Depending on the differentiation stage, osteoblasts change their characteristics. These cells undergo three partly over-lapping stages of osteogenesis^12^. RUNX family transcription factor 2 *(RUNX 2),* a member of the runt transcriptions factor family, is one of the major transcriptional factors during osteoblast lineage and is frequently referred to as “master gene of osteoblast differentiation”^7^. *RUNX2* induces the expression of genes encoding major bone matrix proteins; these genes include collagen type I alpha 1 chain *(COL1A1)*, secreted phosphoprotein 1 (*SPP1;* also known as osteopontin)*,* bone gamma-carboxyglutamate protein *(BGLAP;* also known as osteocalcin), alkaline phosphatase, biomineralization associated (*ALPL*) and other key factors of bone metabolism^13,14^. The capacity to produce mineralized extracellular matrix, regulated by bone matrix protein-associated genes, is an unique feature orchestrated by differentiated osteoblasts and results in accumulation of ionic calcium (Ca^2++^) and inorganic phosphate (P_1_) into extracellular matrix^15–17^.

Staphylococcus species are extremely successful at using diverse mechanisms to damage eukaryotic cells. Surface structures and proteins are identified as powerful virulence factors. Pathogen-associated molecular patterns (PAMP), localized on bacterial cell walls, are one of the major patterns, regulating the pathogenicity of staphylococci by inducing chemokines^18,19^. Another kind of pattern, microbial surface components recognizing adhesive matrix molecules (MSCRAMM), is responsible for the internalization of microorganisms by osteoblasts using e.g. the FnBP-Fn-a5b1 Integrin Pathway^20–24^. Different authors described three groups of secreted toxins by *S. aureus*: (i) membrane damaging toxins which produce pore formation in the cell membrane resulting in cytolytic processes, (ii) toxins that work as superantigens by interfering with receptor functions^25,26^, (iii) enzymes and intracellular- and/or extracellular proteins^27^.

In order to establish a biofilm, *S.* *aureus* and *S.* *epidermidis* produce an Extracellular Polymeric Substance (EPS) that enables the bacteria to settle at the site of infection. The presence of EPS is a physical barrier to external stress and allows microorganisms to proliferate and maturate. The final step, defined as detachment, releases single cells to promote the dissemination of biofilm clusters to distant sites^28,29^. During the development of biofilm, different soluble factors are produced including proteins, eDNA, exopolysaccharide, polysaccharide intercellular adhesin (PIA), carbohydrates, teichoic acids and surfactants^30,31^. The aim of this bacterial coding is to provide a decisive advantage for antimicrobial therapeutics and innate immune defense^32,33^. Biofilms produced by *S.* *aureus* and *S. epidermidis* are very similar concerning structure and function, but show different degrees of virulence^34^. The ability of *S.* *aureus* to adhere on cell surfaces, to invade cells and to produce numerous virulence factors as well as to produce biofilms make *S*. *aureus* an aggressive bacterium, especially for multimorbid and older patients^35–37^.

*S. epidermidis* is a pheno- and genotypically different pathogen of the same genus, which possesses divergent features compared to *S. aureus*^18,38^. It is possible that the lack of aggressive virulence factors like invasins reduces the virulence potential of *S.* *epidermidis*. However, Dapunt et al. demonstrated that *S*. *epidermidis* also has the ability to invade host cells^39^. In addition, although a host immune system has produced antibodies against *S.* *epidermidis* proteins, the host defense may not be efficient. It is reported that exopolymeres, produced by *S.* *epidermidis,* can protect against antibody recognition^18^. As Nguyen et al. reported *S. epidermidis* biofilms are able to reduce the phagocytic function and production of anti-inflammatory cytokines in contrast to their planktonic form^40^.

Despite numerous studies about the interaction of Staphylococcus species, biofilm production and bone infections, further detailed research is needed. Our knowledge concerning the effect of soluble biofilm factors (SBF) and their virulence potential on osteogenic differentiation is limited to few studies^41,42^. Wei-ming et al. reported that *Staphylococci* enterotoxin C2 promotes the osteogenic differentiation in mouse mesenchymal stem cells (msc)^43^ and Wu et al. described a similar effect in human msc. A hyper-mineralization, induced by *Staphylococcus aureus* protein A, in mouse pre-osteoblastic cells was observed by Kavanagh and Claro confirmed that *Staphylococcus aureus* protein A can increase the proliferation of pre-osteoblastic cells^44–46^.

Especially, interindividual differences in cell response, i.e. patient-specific reactions to SBF need to be further elucidated. This is particularly true when there is no direct contact between prokaryotic and eukaryotic cells, but the effects are mediated indirectly. In this study, we investigated primary human osteoblasts (phOB) from seven donors and identified a highly differentiated osteoblast response to biofilm soluble factors from *S.* *aureus* and *S.* *epidermidis.*

## Results

### pH value

To avoid a bias in pH shifting during the SBF setting pH differences between the CO (osteogenic medium (OM) without SBF), and SBF groups, media were adjusted overnight in the CO_2_ incubator and the pH value was checked. No significant differences were measured (data not shown).

### Biofilm viability, characterization and protein concentration of soluble biofilm factors

**Figure 1:**
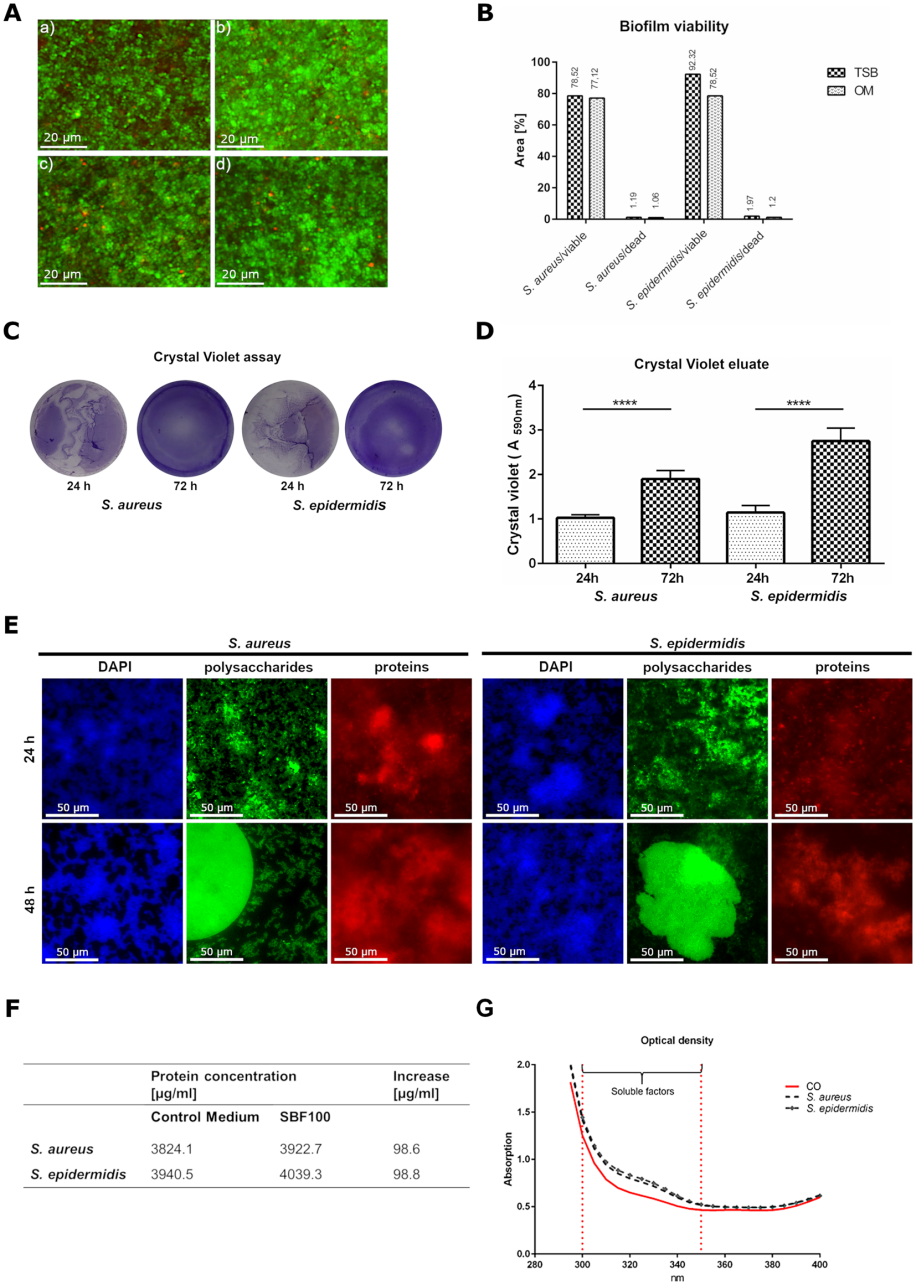
Biofilm characterization and the concentration of SBF in OM. *S.* *aureus* (**A**, a) and *S.* *epidermidis* (**A**, b) after 48 h incubation in 3% TSB. *S. aureus* (**A**, c) and *S.* *epidermidis* (**A**, d) after 48 h incubation in OM. Bacteria with intact cell membranes stained fluorescent green (SYTO 9, ex/em 480/500 nm). Bacteria with damaged membranes stained fluorescent red (propidium iodide ex/em 490/635 nm). The viability of *S. aureus* and *S. epidermidis* in 3% TSB and OM was calculated using ImageJ and revealed comparable results (**B**). The crystal violet assay detects the biomass growing over 72 h (**C**) and the measured crystal violet eluate confirmed these results (**D**). Major components of the biofilm, polysaccharides were stained with Concanavalin A, conjugated with Alexa 488 (ex/em 470/525 nm), proteins were detected by SYPRO Ruby Biofilm Matrix stain (ex/em 450/610 nm) and the DNA was visualized by DAPI (ex/em 358/461 nm) (**E**). The BCA assay revealed a similar increase of nearly 100 µg protein /ml in the OM with SBF from *S*. *aureus* and *S*. *epidermidis* (**F**). The measurement of optical density confirmed the results of the protein concentration (**G**).

Not only the growth behavior but also the viability of both bacterial species seems to be very similar in TSB as well as in OM. To produce realistic results, optimized culture conditions for bacteria and phOB were required. One of the optimal nutrients for *S.* *aureus* and *S. epidermidis* is 3% TSB media, for phOB, the optimal culture medium is osteogenic medium (OM). The biofilm viability of both species in TSB and OM was confirmed by LIVE/DEAD staining. *S.* *aureus* (Fig. 1A, a) and *S. epidermidis* (Fig. 1A, b) cultured in TSB revealed an optimized viability. A slightly decreased growth rate of both bacterial species was detected in OM (*S. aureus* is shown in Fig. 1A, c and *S. epidermidis* in Fig. 1A, d). In conclusion, both, *S.* *aureus* and *S. epidermidis*, showed comparable viability in 3% TSB and OM (Fig. 1B).

In order to prove the biofilm formation of *S. aureus* and *S. epidermidis* crystal violet assay was performed. As shown in Fig. 1C, for both *S. aureus* and *S. epidermidis* a distinct biofilm production was detected after 72 h. In addition, the crystal violet assay revealed the formation of biofilms already during the cultivation period. The crystal violet eluate measured (Fig. 1D) confirmed the increase of biomass. Interestingly, after 24 h the bacteria strains both showed approximately the same quantity of biomass, however, after 72 h the biomass produced by *S. epidermidis* was significantly higher than by *S. aureus*. Furthermore, we visualized major parts of the biofilm composition after 24 h and 48 h. Biofilm Tracer SYPRO Ruby Biofilm Matrix staining revealed protein content and Concanavalin A, conjugated with Alexa 488 show polysaccharides content. DAPI staining localized the bacteria and detected DNA (Fig. 1E). The results of the crystal violet assay confirm the ability of our S. aureus and *S. epidermidis* strains to produce relevant biofilms.

In the next step, SBF were produced in OM and denominated as SBF100% (SBF100). Accordingly, SBF50% (SBF50) was produced by mixing equal parts of SBF100 and OM (1:1). Compared to the control medium (OM without SBF) the protein concentration was measured. Interestingly, using the BCA protein assay, we found a similar increase of nearly 100 µg protein /ml in the OM with SBF of *S*. *aureus* and *S*. *epidermidis,* compared to the control medium (Fig. 1F). These results were confirmed by measurement of the optical density. As seen in Fig. 1G, the increasing absorption values of the optical density measurement correlated directly to that of the protein concentration augmentation.

### SBF affect morphology of primary human osteoblasts

**Figure 2:**
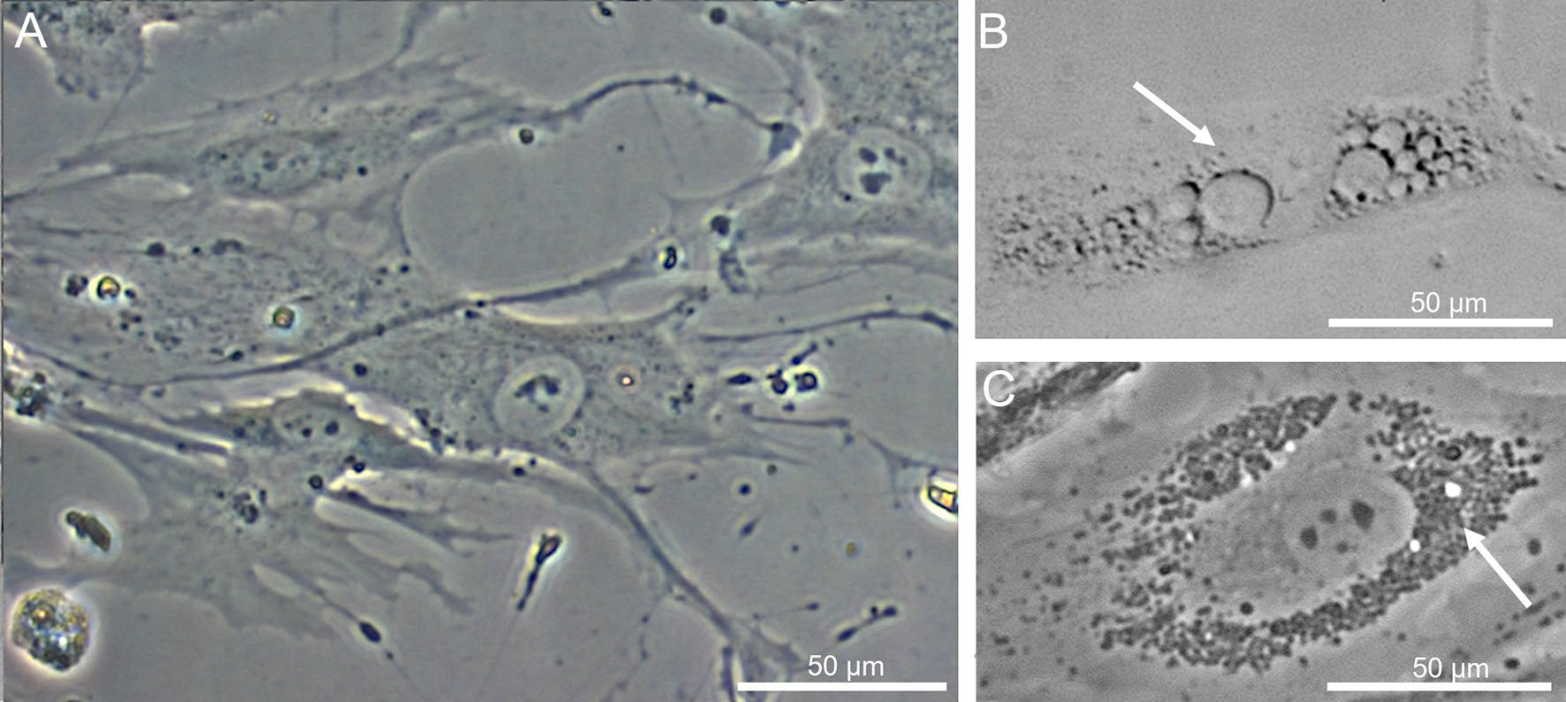
SBF affects the morphology of osteoblasts. phOBs (representative donor) under standard osteogenic culture conditions (**A**). phOBs after treatment with *S. aureus* SBF100 for 72 h (**B**) and *S.* *epidermidis* SBF100 after 72 h (**C**). Image A represent the characteristic appearance of osteoblasts in CO media. The cell membranes of treated phOBs (**B,C**) revealed different types of cell membrane damage, which could be induced by the different soluble factors of biofilm from *S. aureus* or *S.* *epidermidis.*

The osteoblasts of the seven donors included in this study were harvested from cancellous bone of the femoral head, and in preparation of the experiments cultured in osteogenic medium. The osteogenic differentiation was proven by the positive staining of the enzyme alkaline phosphatase (ALP), the transcription factor RUNX2 and the bone protein osteocalcin, which are important markers of the osteogenic phenotype (see Supplementary Fig. S1). For each experimental group, representative microscopic picture is shown (Fig. 2). Standard osteogenic culture conditions showed the characteristic morphology of primary osteoblasts (A), whereas the treatment with SBF100 produced by *S. aureus* (B) or *S.* *epidermidis* (C) for 72 h revealed an impact on morphology of osteoblasts. SBF50 from *S. aureus* and *S.* *epidermidis* are not shown due to the moderate effect on morphology and growth behavior. As depicted in Fig. 2, an alteration of cell membranes is visible in both SBF groups, for both types derived either from *S. aureus* or *S.* *epidermidis*. The cell membranes of treated cells revealed differentiated damage, which could be caused by the different SBF from *S. aureus* or *S.* *epidermidis*.

### SBF of *S. aureus* and *S. epidermidis* inhibits metabolic activity in primary human osteoblasts

**Figure 3:**
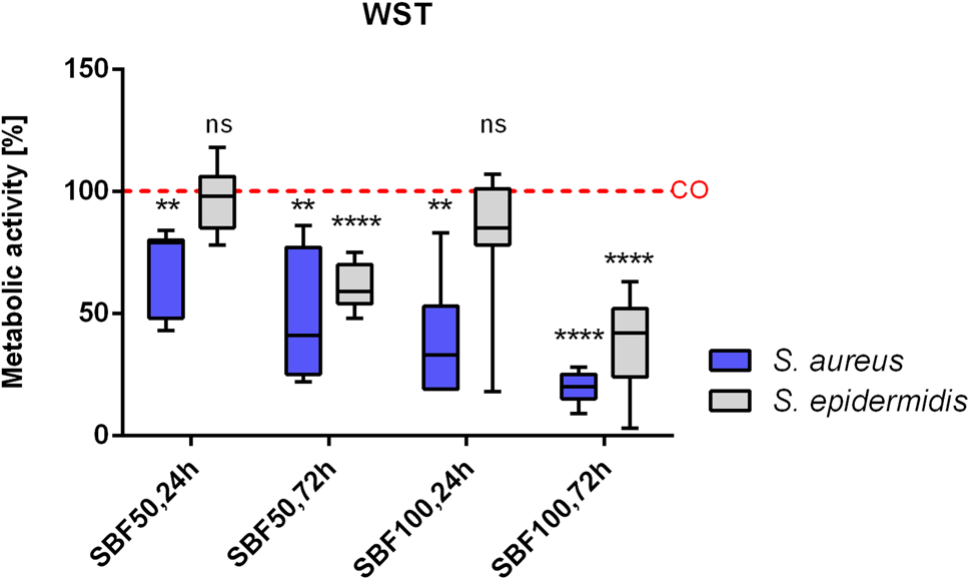
SBF affects the cell metabolism. The partially high impact of the four SBF groups derived either from *S. aureus* or *S.* *epidermidis* (SBF50 24 h; SBF50 72 h; SBF100 24 h; SBF100 72 h) on cell metabolism and viability was measured and the results were compared to the control group. Results are depicted as % of the CO group. The one sample t-test for a null hypothesis (H_0_ = 100) was performed. N = 7. *ns* not significant, *p ≤ 0.05, **p ≤ 0.01, ****p ≤ 0.0001.

To determine the influence of SBF on the cell metabolism, the proliferation rate of osteoblasts was detected after treatment with the four SBF groups derived from *S. aureus* or *S*. *epidermidis* (see Fig. 3: SBF50 24 h; SBF50 72 h; SBF100 24 h; SBF100 72 h). The results are given in comparison with control. A strong inhibitory effect of *S*. *aureus* SBF was observed, dependent on incubation time and different concentrations (SBF50 24 h: p = 0.0174 and 72 h: p = 0.0053; SBF100 24 h: p = 0.0026 and 72 h: p < 0.0001). The SBF of *S.* *epidermidis* revealed slight inhibition in both concentrations after 24 h (statistically not significant), however, after 72 h incubation, osteoblasts responded with a highly statistically significant reduction of metabolic activity (SBF50: p < 0.0001 and SBF 100: p < 0.0001).

### SBF of *S. aureus* and *S. epidermidis* influenced gene expression of osteogenic differentiation in a different manner

**Figure 4:**
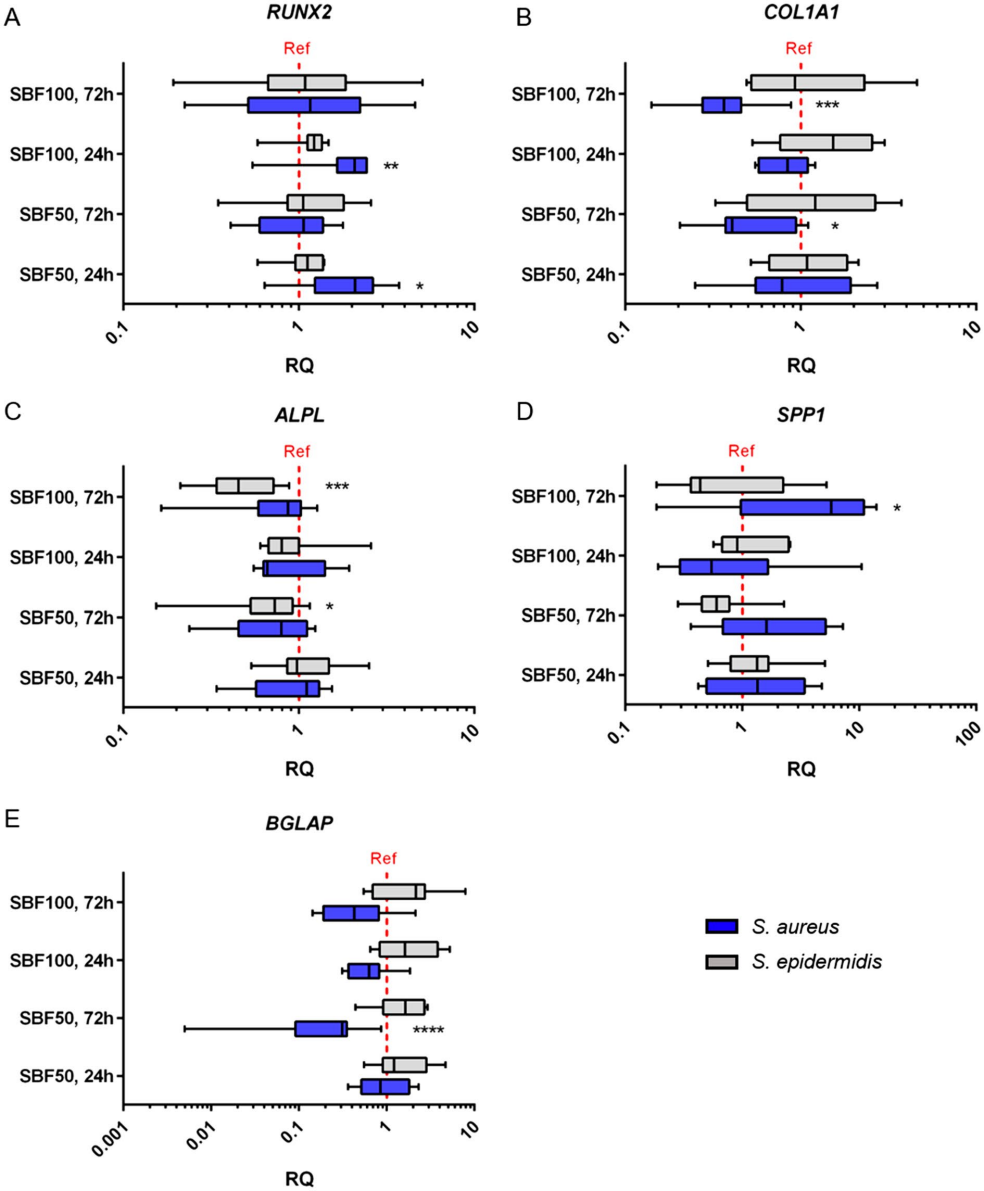
Gene expression changes of genes involved in osteogenic differentiation after stimulation with SBF. qPCR revealed the influence of *S. aureus* or *S.* *epidermidis* soluble factors (SBF50, 24 h; SBF50, 72 h; SBF100, 24 h; SBF100, 72 h) on genes of osteogenic differentiation. CO served as reference (red grid line). *RUNX2*: (**A**); *COL1A1*: (**B**); *ALPL*: (**C**); *SPPL*: (**D**); *BGLAP*: (**E**). The results are presented as relative quantification compared to the CO group. The one sample t-test for a null hypothesis (H_0_ = 1) was done. *p ≤ 0.05, **p ≤ 0.01, ***p ≤ 0.001, ****p ≤ 0.0001.

The transcription factor *RUNX2* is a key factor of bone formation and plays a crucial role in osteoblast development. In order to gain information on the impact of SBF on the differentiation behavior of osteoblasts, qPCR analyses of the four groups from *S.* *aureus* and *S.* *epidermidis* (SBF50, 24 h; SBF50, 72 h; SBF100, 24 h; SBF100, 72 h) were performed. The CO served as reference. The SBF of *S*. *aureus* incubated for 24 h showed a significant upregulation of *RUNX2* in both concentrations (SBF50: p = 0.0196; SBF100: p = 0.0056), whereas an elongated incubation time (72 h) did not statistically significantly affect *RUNX2* expression. The slight influence of *S*. *epidermidis* SBF was observed regarding the SBF100 group, however, these values were not significant (Fig. 4A).

Collagen type I *(COL1A1*) is the most abundant extracellular protein in bone and therefore is essential for bone strength. As our results show, *COL1A1* is downregulated in all *S.* *aureus* groups , particularly after 72 h (SBF50: p = 0.0080; SBF100: p < 0.0001). In contrast to *S*. *aureus, S*. *epidermidis* revealed only moderate alterations of gene expression (Fig. 4B).

Alkaline phosphate (*ALPL*) is an indicator for early osteogenesis and is also associated with matrix maturation and mineralization. Both concentrations of *S.* *epidermidis* SBF showed a significant downregulation after 72 h (SBF50: p = 0.0282; SBF100: p = 0.0004). The 24 h groups do not seem to affect the *ALPL* gene expression. Tendency for moderate alterations in the *S. aureus* groups could be demonstrated by the qPCR (Fig. 4C).

Osteopontin, denominated secreted phosphoprotein 1 by the HGNC *(SPP1*), is a bone protein with multiple functions. The measurement of *SPP1* gene expression using qPCR partially revealed a strong impact. After incubating cells for 72 h with *S.* *aureus* SBF100, we observed a two- to tenfold (p = 0.0292) increase in *SPP1* gene expression, which seems to depend on the SBF concentration applied. The influence of SBF of *S.* *epidermidis* showed a time-dependent downregulation after 72 h, even though, after 24 h only a slight increase was detected (Fig. 4D).

*BGLAP,* also referred as osteocalcin, is synthesized by mature osteoblasts and is responsible for functions including matrix mineralization. *S.* *aureus* derived SBF50 and SBF100 inhibited the *BGLAP* expression after 72 h incubation (SBF50: p < 0.0001, SBF100: no significance). In contrast, SBF from *S.* *epidermidis* in both concentrations revealed a slightly increase, especially after 72 h (Fig. 4E).

### SBF of *S. aureus* and *S*. *epidermidis* affect ALP activity in primary human osteoblasts

**Figure 5:**
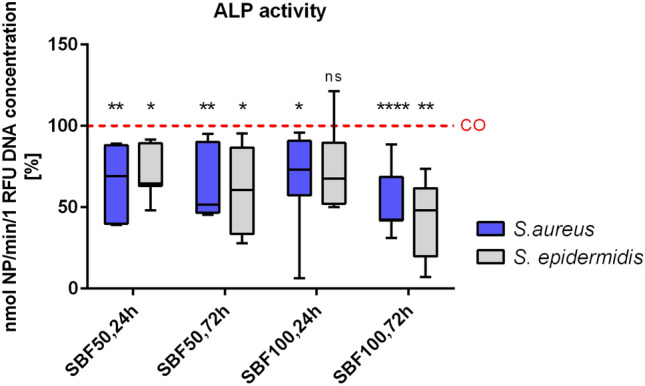
ALP activity after stimulating osteoblasts with SBF. ALP was measured and normalized against total DNA amount. Both concentrations of *S. aureus* and *S.* *epidermidis* SBF impact the ALP activity and resulted in a significant downregulation after 24 h and 72 h, with exception of *S. epidermidis* SBF100, 24 h. *ns* not significant, *p ≤ 0.05, **p ≤ 0.01, ****p ≤ 0.0001.

The ALP enzyme activity was measured and normalized in relation to DNA concentration. The results were compared to the CO group and set at 100% activity***. ***The impact of SBF of both bacteria species was significant: Overall SBF of both bacteria caused a reduction of ALP activity. (*S.* *aureus* SBF50, 24 h: p = 0.0050; SBF50, 72 h: p = 0.0015; SBF100, 24 h: p = 0.0254; SBF100, 72 h: p ≤ 0.0001 and *S*. *epidermidis* SBF50, 24 h: p = 0.0130; SBF50, 72 h: p = 0.0154; SBF100, 24 h ns; SBF100, 72 h p = 0.0024). The results of the ALP enzyme activity test confirmed the impact of SBF on the ALPL gene expression in a time-, concentration- and bacteria-dependent manner (Fig. 5).

### SBF of *S. epidermidis* markedly affects mineralization

**Figure 6:**
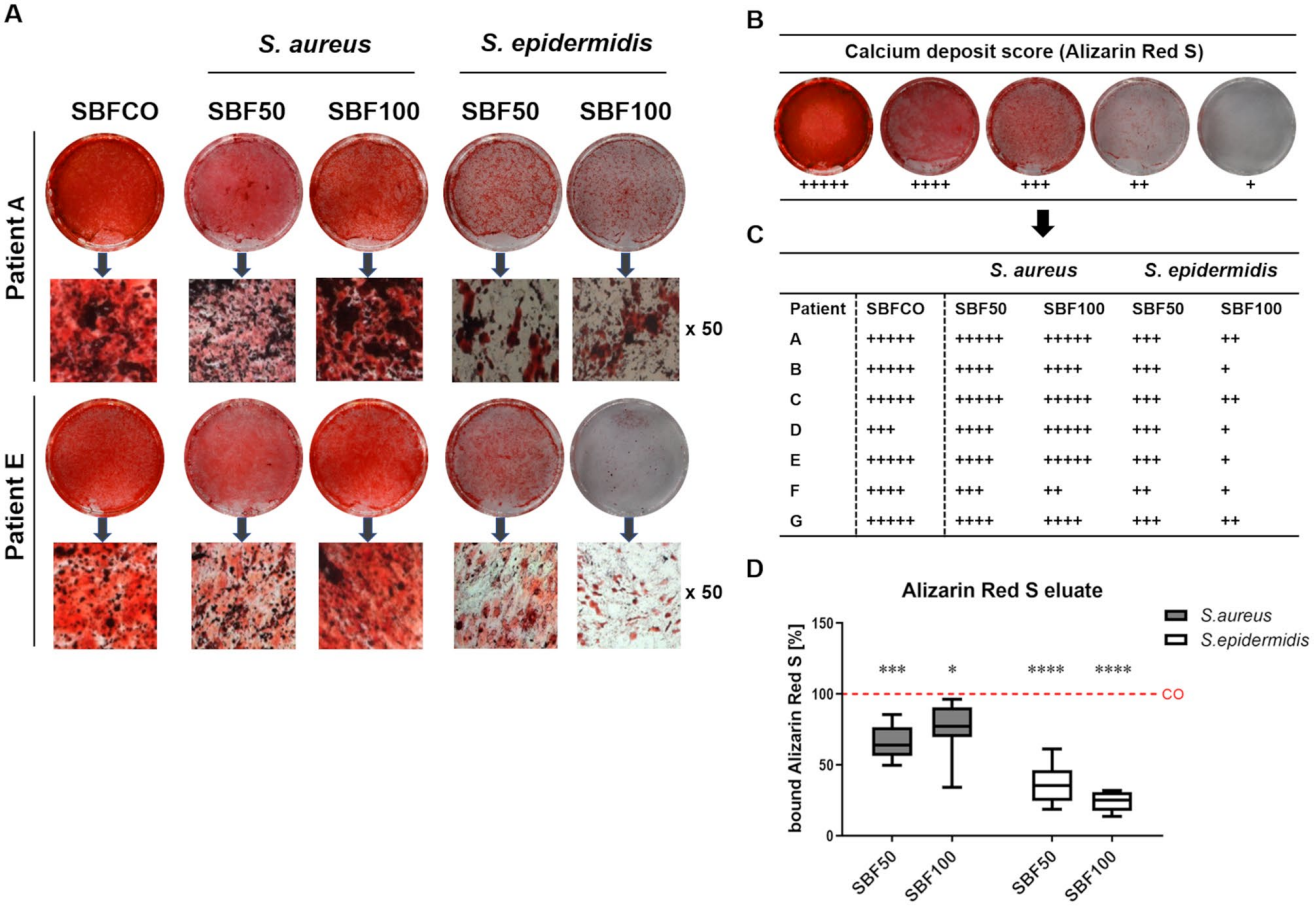
SBF impacts calcium deposits produced by osteoblasts. The calcium deposits were dyed with Alizarin Red S stain after incubation for 28 days, representative images from 2 donors are shown (**A**). Calcium deposit score (**B**). Semi quantitative evaluation of all 7 patients by using the calcium deposit score (**C**). Quantification of bound Alizarin Red by photometric measurement (**D**). *p ≤ 0.05, **p ≤ 0.01, ***p ≤ 0.001, ****p ≤ 0.0001.

The mineralized extracellular matrix is an unique feature of mature osteoblasts and is associated with a number of genes and factors from the osteogenesis process. The calcium deposits were stained using Alizarin Red S and subsequently the quantification of bound Alizarin Red S was performed by photometric measurement after 28 days (Fig. 6). The phOB’s which were cultured in CO medium revealed an abundant strong mineral deposition. The cells from the seven donors, treated with SBF50 or SBF100 from *S.* *aureus,* showed a varying potential for mineralization, whereas the cells treated with SBF50 or SBF100 from *S.* *epidermidis* depicted a high decline in the capability of producing mineralized bone matrix. Representative images from two donors are shown in Fig. 6A. The semi quantitative calculation was carried out by analyzing the calcium deposit score (Fig. 6B) and the detailed results from each of the seven donors are summarized in Fig. 6C. The photometric measurement of bound Alizarin Red S confirms the difference between *S.* *aureus* and *S.* *epidermidis* and the high significance of the results compared to the CO (Fig. 6D).

**Figure 7:**
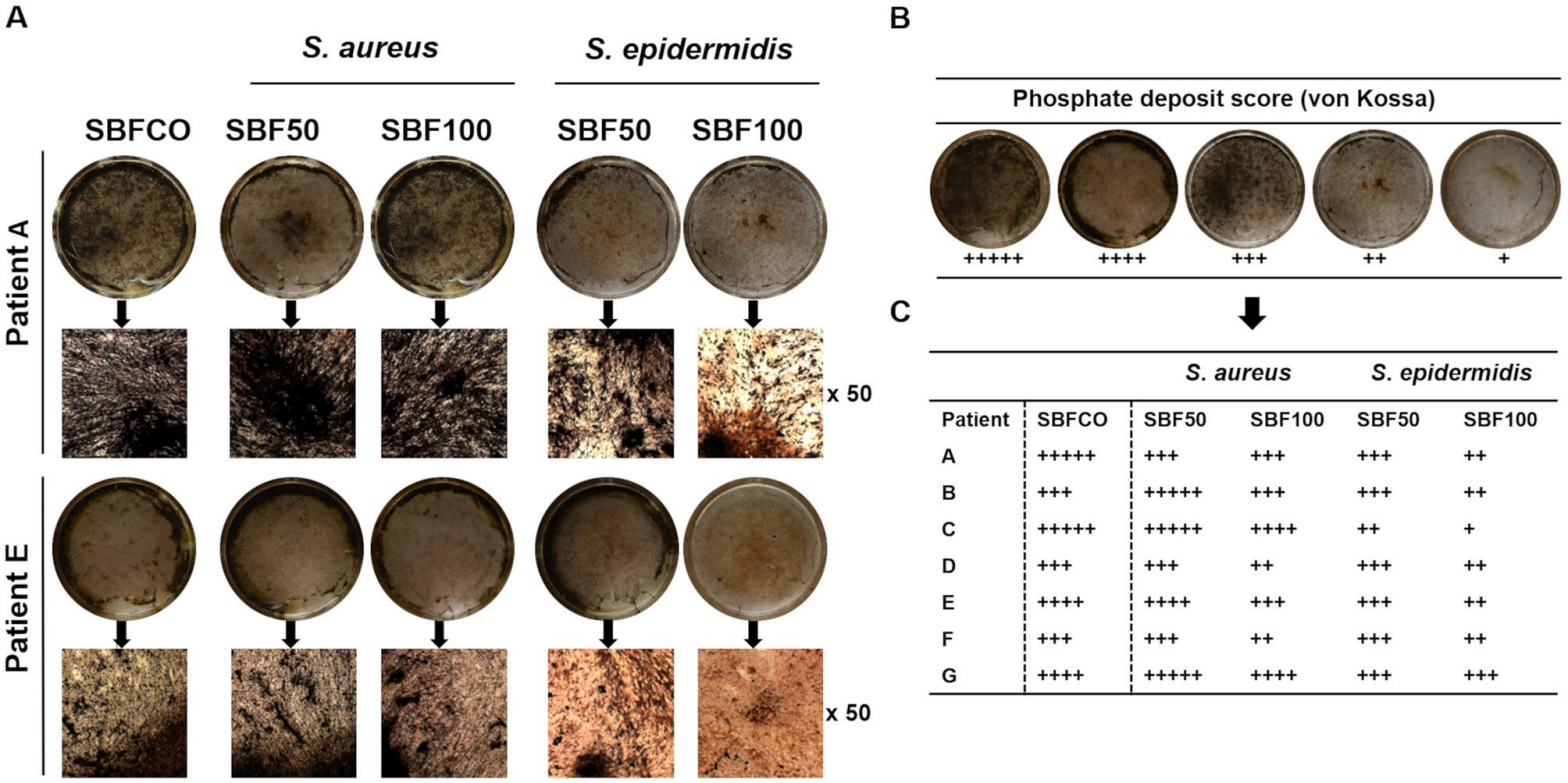
SBF impacts phosphate deposits produced by osteoblasts. The phosphate deposits were dyed with von Kossa stain after incubation for 28 days; representative images from 2 donors are shown (**A**). Phosphate deposits score (**B**). Semi quantitative evaluation of all 7 patients by using the calcium deposit score (**C**).

The phosphate deposits were dyed using von Kossa stain (Fig. 7). The phOBs cultured with CO revealed an abundant strong mineral deposition. Similar to the results of the calcium deposition, *S. aureus* revealed no inhibitory effect on phosphate deposition. Interestingly, the impact of *S.* *epidermidis* on phosphate deposition seems patient-specific and rather moderate. Representative images from two patients are shown in A. The semi quantitatively calculation was carried out by the phosphate deposit score (B) and the detailed results of all seven donors are summarized in C.

## Discussion

Our study is the first to demonstrate the distinct effects of soluble factors from *S. aureus* and *S.* *epidermidis* biofilms on osteogenic differentiation and the mineralization of primary human osteoblasts on different donors. To the best of our knowledge, this is the first time that substantial and patient-specific impact of *S.* *epidermidis* soluble factors on osteogenic differentiation and mineralization have been investigated.

Implant-associated infections are frequently responsible for prosthesis loosening. The treatment is complicated, time consuming and often requires multiple surgical procedures, that become a burden for the patients affected. Approximately 65% of these infections are caused by one of the bacteria investigated. Bacteria have developed several strategies to damage eukaryotic cells: (i) damaging cell wall to invade cells (ii) causing structures in cell wall to work as virulence factors like PAMP or MSCRAMM, (iii) creating toxins that work both as superantigens, and (iv) enzymes and intracellular- and/or extracellular proteins^23,38^. The formation of biofilms is a highly effective mechanism of microorganism as an indirect weapon against antibiotics and the innate immune system^47,48^. With few exceptions, most of our knowledge regarding the influence of bacteria on human primary osteoblasts has been gained from studies, investigating direct interaction between prokaryotic and eukaryotic cells.

As Dal Peraro reported in 2016, pore forming toxins (PFT) are important virulence factors which interact with different cell surface proteins from the host. Recent studies have demonstrated the membrane damage is caused by hemolysins (alpha-, beta-, gamma- and delta-hemolysins), produced by *S.* *aureus*^49,50^. The morphology of phOB after exposure to SBF from *S*. *aureus* or *S. epidermidis* led us to hypothesize that water soluble PFT attacks the osteoblasts. Hence, we primarily observe severe membrane damage to cells treated with SBF produced by *S.* *aureus*. It may be possible, that the effects of SBF derived from *S. epidermidis* is delayed in time or that the toxins in the *S*. *epidermidis* SBF are less virulent. In our opinion these ideas are worth further investigations.

In accordance with other studies^41,42^, soluble biofilm factors showed a direct effect on the metabolic activity of human primary osteoblasts, which seems to depend on patient demographics, medical history, incubation time, bacteria species and the concentration of soluble biofilm factors. Preliminary tests carried out with SBF and the supernatant from planktonic cultured bacteria confirmed the results of Ward et al.^42^ and other authors that SBF are more toxic than supernatants from planktonic culture bacteria. For this reason, and the relevance of biofilms for implant-associated infections we decided to focus the study on soluble biofilm factors. The decision to use SBF at the concentration of 50% and 100% based also on preliminary tests. After treatment with SBF 50% and SBF100% the osteoblasts responded with different alterations. However, after exposition with SBF 10%, the results measured partially showed a marginal effect or a slight increase of proliferation (data not shown).

The mechanism of osteogenic differentiation and matrix mineralization is well known. Numerous studies explain the regulation by the major factors involved in these processes, e.g. *RUNX2, ALPL,*and *BGLAP*^13,51^. The present study exhibits a new information: the significant upregulation of *RUNX2* after 24 h. This corresponds to a prompt response to soluble biofilm factors derived from the bacteria species investigated. Considering the downregulation of “-genes involved in matrix mineralization”-, in particular *ALPL*, *BGLAP* and *COL1*, the results of this study support the idea of an early onset of the impacts of soluble biofilm factors observed in this experimental set up. However, we identified substantial patient-specific difference in responds to the toxic stimuli applied (see Supplementary Fig. S2). In the future our study group aims at investigating even larger cohorts. This may deliver further insights into these effects. Although, cultivation of well differentiated and undoubtedly characterized primary osteoblasts from human specimen is challenging and time-consuming, we think this effort is worthwhile as these cells more accurately depict individual responses to infection. Interestingly, despite these results and a significant reduction of ALP activity we could observe a remarkable calcium deposition after 28 days, which was completely unexpected. In contrast, few studies reported a total lack of calcium deposition after exposure to soluble biofilm factors produced by *S*. *aureus.* Furthermore, these studies used different bacterial strains of *S*. *aureus* and the cell types used are heterogeneous (e.g. mouse osteoblasts, human osteoblast like cell lines)^37,45^. In primary human osteoblasts a donor- and concentration dependent calcium deposition was observed.

Remarkably, we measured a reduction of bound Alizarin Red after treatment with diluted SBF from *S. aureus*, whereas a slight augmentation after exposure to undiluted SBF was observed. Hence, there is a strong evidence that the osteoblast’s response to soluble biofilm factors of *S*. *aureus* may also be regulated by the concentration of toxins. Recently it has been reported that osteogenesis can be promoted by the superantigen enterotoxin C2, which is synthesized by *S. aureus*^46^. Kavanagh et al. observed a 3D culture where a hyper-mineralization was induced by Staphylococcus aureus protein A^45^. More recently, Tomizawa et al. presented an osteomyelitis mouse model. The results show, that *S.* *epidermidis* (RP62A) is able to stimulate a pro-inflammatory environment, which might cause an incomplete osseous integration of an implant^52^. Our findings regarding soluble biofilm factors from *S.* *aureus* support the idea that virulence factors can boost the osteogenic processes. However, if concentrations of soluble factors exceed certain levels, these effect show an inverse pattern.

Considering the mechanisms in the late maturation stage of osteoblasts, a high synthesis rate of alkaline phosphatase is required to hydrolyze pyrophosphate into inorganic phosphate (P_I_)^53^, which promotes hydroxyapatite crystal growth^54^. The decrease of phosphate deposition in this study illustrates a potential correlation between the reduction in the *ALPL* gene expression on a mRNA level and a greatly reduced ALP activity on a protein level, with a seemingly stronger influence of *S*. *epidermidis* than *S.* *aureus*. As demonstrated in our patient cohort, these effects appear to be heterogeneous. Furthermore, the results illustrate that calcium- and phosphate- deposition is concentration-, patient-, and bacteria dependent.

The pathogenic mechanisms of *S. epidermidis* on osteoblasts seem to be different as compared to *S. aureus*. The lack of *S. epidermidis’* aggressive virulence factors appear to reduce its pathogenic potential. Nevertheless, *S. epidermidis* is clinical highly relevant and the incidence of infections caused by *S.* *epidermidis* in our field increase. In addition to its ability to colonize biomaterials and to form biofilms, *S. epidermidis* is one of the major causes of low-grade infections. Although, this has been known for few years, this has not attracted sufficient attention in orthopedics, outside specialized centers. This may explain the substantial lack of detailed literature regarding the impact of *S. epidermidis* on osteogenic differentiation. Responsible for approximately 20–30% of orthopedic implant-related infections and up to 50% of late-developing infections^55^, *S*. *epidermidis* infections have become a clinically highly relevant and serious problem. Additionally, diagnosis and therapy is technically increasingly challenging because of demographic change. Older patients show a higher risk of surgical site infections frequently due to comorbidities as osteoporosis, diabetes or other skeletal diseases^55^. Our study demonstrates the major impact of *S. epidermidis* on osteogenic differentiation, especially the deficient calcium deposition, which is an unexpected but interesting new insight. The reasons why soluble factors of *S.* *epidermidis* biofilm impact the calcium deposition more than *S. aureus* has not been clarified yet. Hence, more detailed investigations with larger cohorts are required. Despite the reduced metabolic activity measured a part of the osteoblasts seemed to be able to produce a calcium- and phosphate deposition after treatment with SBF. The authors hypothesize that osteoblasts of different maturation stages are differentially able to cope with toxic stimuli of the SBF.

Due to the fact that our study was carried out using human primary osteoblasts from seven donors, it is difficult to compare it with other studies using murine or human cell lines. In our opinion using primary human osteoblasts from multiple donors is an advantage of this study as one may hypothesized that these cells behave similar to those in human tissue. However, the data presented is of preclinical character and has yet to be further investigated. Furthermore, due to the heterogeneity of donors (age, gender, previous illness), it is problematic to compare individual patients. As we used primary human cells from different donors, the variance of individual data only reflects interindividual differences in donor cell response that is characteristic for complex biological processes. Hence, the authors emphasize the necessity to evaluate larger numbers of patients and an elongated exposition time.

Our study holds some limitations. First, we did not use isolated clinical strains. Second, we did not evaluate the biofilm proteome. Third, an expansion of measurement points should provide more precisely and detailed results of cell response after treatment with soluble factors.

## Conclusion

In the present study, we show that soluble factors derived from both *S.* *aureus* and *S*. *epidermidis* biofilms reduce cell proliferation, metabolism and change the expression of “genes and proteins involved in matrix mineralization”. To our knowledge, this study is the first to demonstrate a significant reduction of calcium deposition due to soluble factors of *S*. *epidermidis* biofilm. Additionally, the effects of soluble factors from *S*. *aureus* or *S*. *epidermidis* biofilm measured seem to be strongly dependent on : (i) the concentration: high levels of soluble factors are toxic; lower concentrations seem to have the contrary effect and seem to increase osteogenic differentiation. (ii) Similarly increasing exposition time to SBF causes toxic effects, short exposition time to SBF seems to increase osteogenic differentiation. (iii) The effects observed are patient-specific. (iv) Distinct differences between the effects of soluble factors from *S. aureus* and *S.* *epidermidis* were observed.

Our findings identify major differences in the virulence potentials of soluble biofilm factors from *S*. *aureus* and *S*. *epidermidis*. Therefore, these new findings confirm support the hypothesis that soluble biofilm factors affect osteogenic processes substantially, particularly when there is no direct interaction between bacteria and osteoblast. These mediator-driven effects are comparable to effects induced by physical bacteria to cell interactions.

## Material and methods

The study was performed with the approval of the Local Ethics Committee of the University Hospital of the Technical University of Munich (1307/05). Written informed consent was obtained from each patient. All experiments and methods were performed in compliance with the relevant guidelines and regulations and in accordance with the ethical standards of the Helsinki Declaration.

### Culture conditions and cell isolation

All cell cultures were maintained at 37 °C in a 100% humidified atmosphere containing 5% CO_2_. Primary human osteoblasts (phOB) are cultured in osteogenic medium (OM) containing Dulbecco’s minimum essential medium, 15% fetal bovine serum, 2 mM l-glutamine, 0.8% MEM vitamins, 2.8 µM ascorbic acid (all obtained from Sigma Aldrich, Deisenhofen, Germany)^56^ and was changed twice a week (unless otherwise noticed).

Cells of seven donors were included in this study, the average age was 53 ± 14 years (age range 36–67 years) (five females, two males), for detailed characteristics see Supplementary Table S1. phOBs were gained from cancellous bone of the femoral heads obtained from bone resection during hip arthroplasty. phOBs were isolated using the explant method as described previously in detail^57^. Briefly, small cancellous bone pieces were extracted from the femoral heads, cultured in OM and after reaching 80% confluence cells were amplified. An alkaline phosphatase test with NBT/BCIP according to the manufacturer’s instructions (Roche, Mannheim, Germany) was proceeded in order to confirm the osteogenic phenotype of the cultured cells. Cells at passage number 4–9 were used for the experiments.

### Production of soluble biofilm factors (SBF)

**Figure 8:**
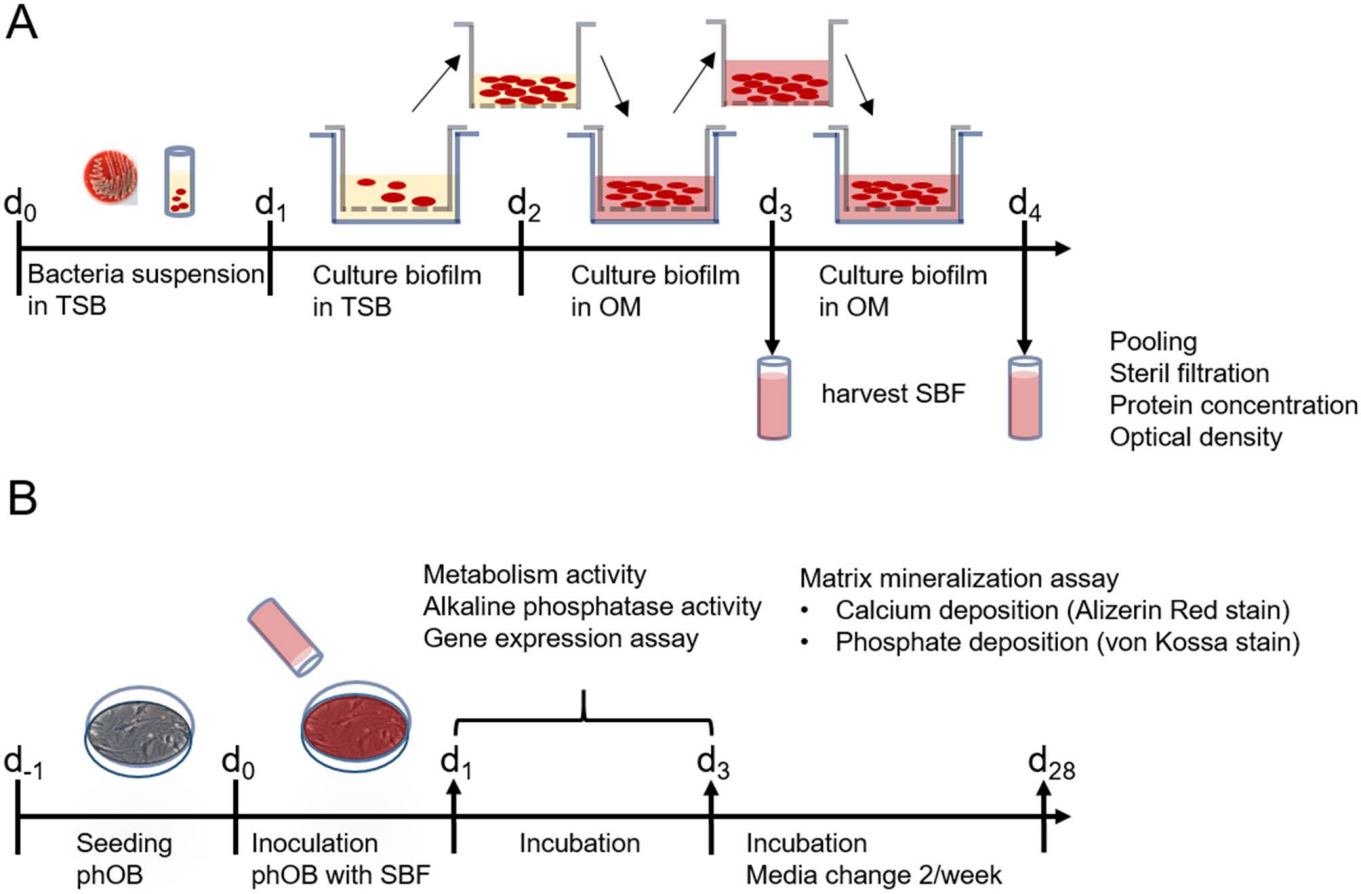
Preparation of soluble biofilm factors (SBF). Illustration of the single steps along the timeline. *TSB* tryptic soy broth, *OM* osteogenic medium (**A**). Experimental design. Illustration of the experimental steps and endpoints along the timeline. *phOB* primary human osteoblasts (**B**).

*Staphylococcus aureus* subsp. *aureus* (ATCC 25923) were streaked onto Columbia Agar with 5% Sheep Blood and incubated overnight at 37 °C to obtain single colonies. Four of them were scraped off the agar plate, suspended in 3% Tryptic soy broth (TSB) and incubated for 5 h at 37 °C to reach the exponential growth phase (OD600 2–3). Bacteria suspension was diluted with TSB to reach an OD600 of 0.3–0.5. An insert (Thin Cert, Greiner BioOne, Austria; pore size 0.4 µm) was placed in a well of a 6 well plate, containing 3 ml TSB and subsequently 2 ml of the bacteria suspension was filled into the insert. After 24 h aerobic incubation at 37 °C a biofilm became visible. The insert was transferred to new wells containing 3 ml OM. After 24 h and 48 h incubation (37 °C, aerobic) the osteogenic differentiation medium containing soluble biofilm factors was harvested, filtrated (0.2 µm), pooled and denominated as soluble biofilm factors 100% (SBF100). Accordingly, SBF50 was produced by mixing equal parts of SBF100 and OM (dilution 1:1). SBF was frozen at − 20 °C until further use. OM without biofilm soluble biofilm factors served as control (CO). The pH value of the media was controlled. Under same conditions, SBF for *Staphylococcus*. *epidermidis* (ATCC 35984) was produced (Fig. 8).

### Biofilm characterization

In order to proof the biofilm viability in both TSB and OM a LIVE/DEAD staining (BacLight, Bacterial Viability Kit, Thermo Fisher Scientific, Germany) was proceeded according to the manufacture’s protocol. Briefly, bacteria with intact cell membranes stained fluorescent green (SYTO 9, ex/em 480/500 nm) whereas bacteria with damaged membranes stained fluorescent red (propidium iodide ex/em 490/635 nm). A mixture of SYTO 9 and propidium iodide (1:1 in 0.3% DMSO) was added to the biofilm and incubated for 15 min in the dark. The exposure time was 60 ms.

To determine the amount of biomass produced by sessile growing bacteria a crystal violet assay, described by Stepanovic et al.^58^ was performed. After growing for 48 h the biofilm was carefully washed three times with PBS, then fixed with 100% methanol and air dried. The biofilm was stained using 0.5% (w/v) crystal violet (CV) (Sigma Aldrich, Germany) for 10 min, the excess dye was removed by running tap water. Subsequently the dye bound to adherent bacteria was solubilized using 33% acetic acid (v/v) and the OD was quantified at 570 nm using a microplate reader.

The different components of the biofilms produced were visualized using fluorescent dyes. Polysaccharides were detected with Concanavalin A, conjugated with Alexa 488 (Thermo Fisher Scientifc, Germany) (50 µg/ml) for 5 min. Film Tracer SYPRO Ruby Biofilm Matrix Stain (Thermo Fisher Scientific, Germany) was used for detecting the proteins according to the manufacturer’s instructions. DNA was stained using Mounting Media DAPI stain (Dianova, Hamburg, Germany). Wavelengths used for Alexa 488 were ex/em 470/525 nm, for SYPRO Ruby ex/em 450/610 nm and for DAPI ex/em 358/461 nm.

### Protein concentration and optical density of soluble biofilm factors

Protein concentrations of the harvested SBF and the control medium was determined using the BCA (bicinchoninic acid) Protein Assay from Pierce (Thermo Fisher Scientific, USA) according to the manufacturer’s protocol. The concentrations were calculated from an appropriate standard curve, using bovine serum albumin. In order to detect differences by optical absorption of the harvested SBF and the control medium, photometric absorption at a range of 280 to 400 nm after 48 h was measured (Multiscan GO, Thermo Fisher Scientific).

### Metabolic activity

For metabolic viability, cells were seeded at a density of 1.2 × 10^4^/cm^2^ for 24 h and 1.0 × 10^4^/cm^2^ for 72 h of incubation in OM. After 24 h, the OM was replaced by CO, SBF 50 or SBF 100. Metabolic activity was measured using a WST-1 Assay (water-soluble tetrazolium salt) (Roche Applied Science, Germany). After incubation with SBF100, SBF50 and the CO (OM without SBF), WST-1 reagent (1/10 volume) was added to each well, incubated for 3 h at 37 °C, and measured at 450 nm using a micro plate reader (Multiskan Ascent, Thermo Fisher Scientific). The viability of the cells incubated in SBF50 and SBF100 is calculated and depicted in relation to the CO.

### Alkaline phosphatase (ALP) activity assay

Under alkaline conditions, ALP can catalyze the hydrolysis of p-nitrophenol phosphate into phosphate and p-nitrophenol. The release of p-nitrophenol per minute was measured and was related to the amount of alkaline phosphastase. Cells were seeded at a density of 1.2 × 10^4^/cm^2^ for 24 h and 1.0 × 10^4^/cm^2^ for 72 h in OM. After 24 h the medium was replaced by CO, SBF50 or SBF100. After incubation for 24 h and 72 h, cell lysates were produced by adding Triton 1% (v/v) and ALP activity measurement was performed using Ecoline Alkaline phosphate activity kit (DiaSys, Holzheim, Germany) according to the manufacturer’s instructions. After calculating the quantity of ALP in relation to a suitable standard curve, ALP levels were normalized with the DNA concentration measured using the CyQuant Direct Proliferation Assay (Thermo Fischer Scientific, USA). The ALP activity was reported as nM/min/DNA concentration.

### Matrix mineralization assay (Alizarin Red S and von Kossa stain)

Cells were cultured with CO, SBF50 or SBF100 (all media were supplemented with 10 mM ß-glycerophosphate, Sigma Aldrich, Germany) for 28 days and media were changed twice a week. Cells were washed with PBS 0.1 M and fixed with 4% paraformaldehyde in PBS. Calcium deposits were assayed by Alizarin Red S staining as followed: After air-drying, 0.5% Alizarin Red solution (pH 4, g/v) (Sigma Aldrich, Germany) was added, incubated for 10 min and subsequently the unbound dye was completely washed away. In order to the quantify Alizarin Red S signal, the dye was eluted for 15 min at room temperature using a solution of 20% methanol, 10% acetic acid and 70% distilled water and measured at 450 nm. The phosphates were stained by von Kossa dye (Sigma Aldrich, Germany) applying a 3% silver nitrate solution in the dark for 30 min, followed by three repeated washing steps and incubation with sodium carbonate-formaldehyde solution to develop the color (dark brown). The two stains were evaluated both macroscopically and microscopically; and then evaluated semi quantitatively by using the calcium- or phosphate deposition score.

### Quantitative real-time polymerase chain reaction (qPCR)

Cells were seeded at a density of 1.2 × 10^4^/cm for 24 h and 1.0 × 10^4^/cm and for 72 h. After 24 h, media were replaced by CO, SBF50 or SBF100. RNA isolation was performed using RNeasy Mini Kit (QIAGEN, Hilden, Germany) according to the manufacturer’s instructions. RNA was eluted in RNase-free water and the quantification and quality control was performed using Nanodrop (Thermo Fisher Scientific). Afterwards RNA was transcribed into cDNA according to the manufacturer’s instructions of Qiagen QuantiTect Reverse Transcription Kit. Taqman real-time PCR reactions were carried out by means of the Applied Biosystems StepOnePlus Real-Time PCR System (Live Technology, Carlsbad, USA). Samples were analyzed in triplicates, in which CO served as reference. Relative gene expression was determined using GAPDH as endogenous control and the 2^−ΔΔct^ method. The primer sequences, i.e. Taq Man Assay IDs used are listed in Supplementary Table S2.

### Statistical analysis

Data are presented as mean ± SD. Depending on the available quantity of cells or RNA, two or three independent experiments and 3–7 technical replicates were processed. Statistical analysis was performed using GraphPad Prism (version 6.0; GraphPad Software Inc., San Diego, CA, USA). Using the one sample t-test results were calculated against the null hypothesis (gene expression H_0_ = 1, all other H_0_ = 100%). p < 0.05 were considered statistically significant.

## Supplementary Information

Supplementary Information.
