# Supplementary material for: Patient-specific effects of soluble factors from Staphylococcus aureus and Staphylococcus epidermidis biofilms on osteogenic differentiation of primary human osteoblasts
Source: Sci Rep. 2021 Aug 26;11:17282. doi: 10.1038/s41598-021-96719-4 (PMC8390505; doi:10.1038/s41598-021-96719-4)
Supplement: Supplementary file 1 — Supplementary Information. [file 41598_2021_96719_MOESM1_ESM.pdf]

Supplementary Information:

Patient-specific effects of soluble factors from *Staphylococcus aureus* and *Staphylococcus epidermidis* biofilms on osteogenic differentiation of primary human osteoblasts

Jutta Tübel[1*], Elisabeth Maier[1], Magdalena Jegen[1], Carmen Marthen[1], Andreas Obermeier[1], Alexander T. Haug[1], Jochen Schneider[2], Rainer Burgkart[1]

Figure S1:

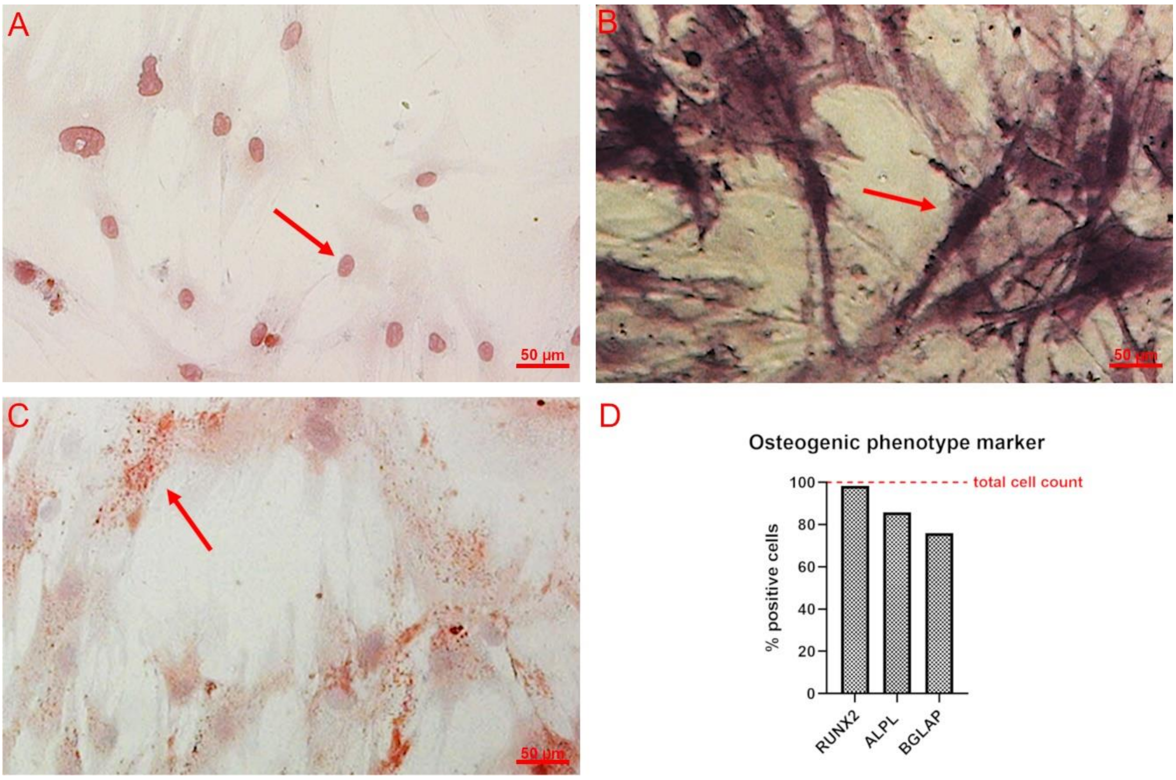

Fig. S1 The osteogenic phenotype of the primary osteoblasts used were confirmed by the positive stain of RUNX2 (A), alkaline phosphatase (B) and osteocalcin (C) and the semi quantitative evaluation is summarized in D.

Figure S2:

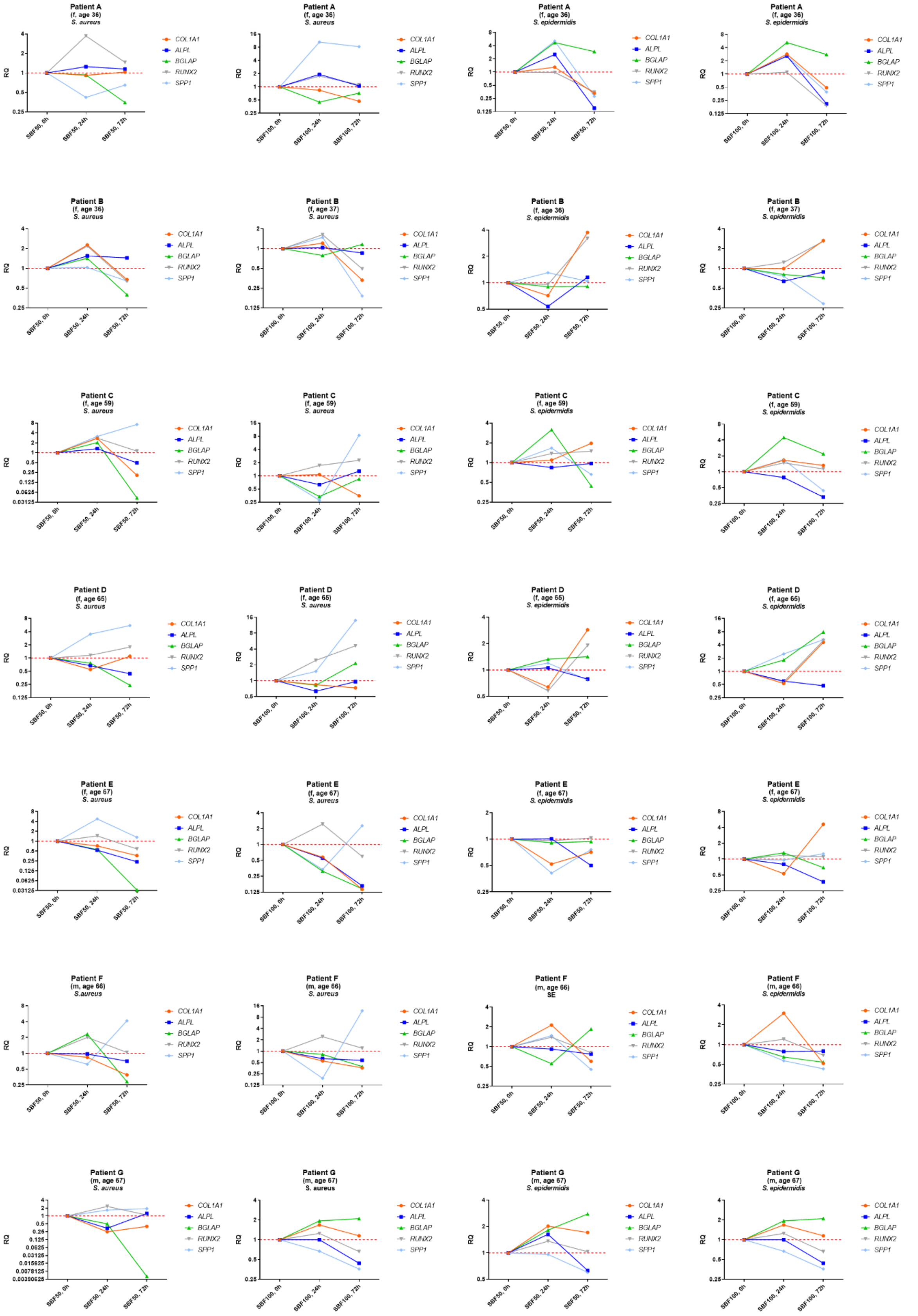

Fig. S2 Gene expression results of *COL1A1*, *ALPL*, *BGLAP*, *RUNX2* and *SPP1*, shown for individual donors.

Table S1 Donor characteristics

Patient	Age	Gender
**A**	36	female
**B**	37	female
**C**	59	female
**D**	65	female
**E**	67	female
**F**	66	male
**G**	67	male

Table S2 Primer ID

Gene Symbol	Gene Name	TaqMan Assay ID
ALPL	alkaline phosphatase, liver/bone/kidney	Hs01029144_m1
BGLAP	bone gamma-carboxyglutamate protein	Hs01587814_g1
COL1A1	collagen type I alpha 2 chain	Hs01028970_m1
RUNX2	RUNX family transcription factor 2	Hs01047973_m1
SPP1	secreted phosphoprotein 1	Hs00959010_m1
GAPDH	glyceraldehyde-3-phosphate dehydrogenase	Hs99999905_m1
